# Incidence of Electrographic Seizures in Patients With COVID-19

**DOI:** 10.3389/fneur.2021.614719

**Published:** 2021-02-04

**Authors:** Brandon L. Waters, Andrew J. Michalak, Danielle Brigham, Kiran T. Thakur, Amelia Boehme, Jan Claassen, Michelle Bell

**Affiliations:** ^1^Department of Neurology, Columbia University Irving Medical Center, Neurological Institute, New York Presbyterian Hospital, New York, NY, United States; ^2^Department of Neurology and Epidemiology, Sergievsky Center, Columbia University Irving Medical Center, New York, NY, United States

**Keywords:** COVID-19, coronavirus, EEG, seizure, status epilepticus

## Abstract

Critical illness and sepsis are commonly associated with subclinical seizures. COVID-19 frequently causes severe critical illness, but the incidence of electrographic seizures in patients with COVID-19 has been reported to be low. This retrospective case series assessed the incidence of and risks for electrographic seizures in patients hospitalized with COVID-19 who underwent continuous video electroencephalography monitoring (cvEEG) between March 1st, 2020 and June 30th, 2020. One hundred and twenty-two patients were initially identified who resulted SARS-CoV-2 nasopharyngeal RT-PCR swab positivity with any electroencephalography order placed in the EMR. Seventy-nine patients met study inclusion criteria: age ≥18 years, >1 h of cvEEG monitoring, and positive SARS-CoV-2 nasopharyngeal swab PCR. Six (8%) of the 79 patients suffered electrographic seizures (ES), three of whom suffered non-convulsive status epilepticus. Acute hyperkinetic movements were the most common reason for cvEEG in patients with ES (84%). None of the patients undergoing cvEEG for persistent coma (29% of all patients) had ES. Focal slowing (67 vs. 10%), sporadic interictal epileptiform discharges (EDs; 33 vs. 6%), and periodic/rhythmic EDs (67 vs. 1%) were proportionally more frequent among patients with electrographic seizures than those without these seizures. While 15% of patients without ES had generalized periodic discharges (GPDs) with triphasic morphology on EEG, none of the patients with ES had this pattern. Further study is required to assess the predictive values of these risk factors on electrographic seizure incidence and subsequent outcomes.

## Introduction

Coronavirus disease 2019 (COVID-19) has been associated with several neurological syndromes (1). The existing literature suggests that the incidence of seizures in COVID-19 patients is relatively low. Small case series have described new-onset electrographic seizures (2, 3)—both clinical and subclinical—and status epilepticus (4) in patients with COVID-19, but a larger retrospective review including 304 COVID-19 patients did not find any cases of clinical seizures (5). The patients in the larger study were not monitored with continuous video electroencephalography (cvEEG), so it is possible that these patients had electrographic subclinical seizures. Electrographic subclinical seizures are of particular concern in COVID-19 patient since up to one fifth of patients with COVID-19 are critically ill (6); subclinical seizure activity can be seen on cvEEG in as many as 19% of critically ill patients in medical intensive care units [ICUs; (7, 8)]. The goal of this study is to describe the results of 257 EEG days across 79 patients as well as to identify the incidence of, and risk factors for, detecting seizures on cvEEG.

## Methods

### Subjects

In this retrospective case series, we evaluated patients who were hospitalized at four New York Presbyterian Hospitals—Columbia University Irving Medical Center (CUIMC), Morgan Stanley Children's Hospital, Allen Hospital, or Lawrence Hospital—from March 1st 2020 to June 30th 2020 and fulfilled the following inclusion criteria: (1) age ≥18 years old; (2) a positive SARS-CoV-2 nasopharyngeal Real-Time Reverse Transcriptase PCR swab; and (3) connection to cvEEG during the same hospitalization for COVID-19 for longer than 1 h. Patients were excluded if the cvEEG was performed during a separate hospitalization. Patient information and cvEEG results were obtained from review of the electronic medical record. This study was approved by the institutional review board at CUIMC.

### EEG Placement and Monitoring

Electrode placement followed the international 10–20 system. EEG was recorded using a digital video EEG bedside monitoring system (Xltek; Natus Medical) EEGs were interpreted by board-certified clinical neurophysiologists/epileptologists and reported using the American Clinical Neurophysiology Society's Standardized Critical Care EEG Terminology (9).

### Procedures and Statistics

Major indications for EEG monitoring were acute hyperkinetic movements, altered mental status, and persistent coma. Acute hyperkinetic movements were either focal or generalized. Altered mental status was defined as acute encephalopathy, either fluctuating, declining or stable, with level of alertness higher than coma. Patients with persistent coma connected to cvEEG were typically those who remained clinically comatose despite the withdrawal of sedating or anesthetic medications. Other indications for EEG monitoring were acute therapeutic temperature management protocol in post-cardiac arrest patients. All cases of acute brain injury were diagnosed clinically or radiographically. The Salzburg criteria were used for classifying non-convulsive status epilepticus, as applied ultimately by fellowship-trained epilepsy faculty at Columbia University Medical Center (10). R version 3.6.1 was used to calculate descriptive statistics, including frequencies, median, interquartile range (R Foundation for Statistical Computing, Vienna, Austria).

## Results

A total of 79 patients met the inclusion criteria comprising 257 days of EEG recording (Table 1). The most common indication for cvEEG was hyperkinetic movements, followed by altered mental status and persistent coma. Six (8%) patients had seizures captured on EEG, with 3 of these meeting criteria for non-convulsive status epilepticus. Five of the 6 patients with electrographic seizures were monitored due to concern for seizure in the setting of hyperkinetic movements; the sixth was monitored for fluctuating mental status in the setting of acute intracranial hemorrhage. None of the patients monitored for persistent coma or post-cardiac arrest (*N* = 27) had electrographic seizures (Table 1).

**Table 1:** Cohort characteristics.

**Clinical characteristics**	**All patients (*n =* 79)**	**Seizures on EEG (*n =* 6)**	**No seizures on EEG (*n =* 73)**
Median age (IQR)	64 (57, 70)	61 (57, 66)	64 (57, 71)
Number of female patients (%)	25 (31.6)	4 (66.7)	21 (28.8)
Total days of recording	257	47	210
Median days of recording per patient (IQR)	2 (1, 3)	6 (5, 12)	2 (1, 3)
Number of seizure days (percentage of total days of recording)	11 (4.3)	11 (23.4)	x
History of seizure (%)	7 (8.9)	3 (50.0)	4 (5.5)
History of chronic brain disease (%)	24 (30.4)	4 (66.7)	20 (27.4)
ICU admission (%)	64 (81.0)	4 (66.7)	60 (82.2)
Acute brain injury (%)	27 (34.2)	3 (50.0)	24 (32.9)
Anoxic/Hypoxemic injury	4 (5.1)	0 (0)	4 (5.5)
Acute ischemic stroke	11 (13.9)	0 (0)	11 (15.1)
Acute intracranial hemorrhage	12 (15.2)	2 (33.3)	10 (13.7)
PRES	1 (1.3)	0 (0)	1 (1.4)
Worsening vasogenic edema	1 (1.3)	1 (16.7)	0 (0)
**Primary reason for admission (%)**			
COVID-19 only	60 (75.9)	2 (33.3)	58 (79.5)
Neurologic disease	7 (8.8)	2 (33.3)	5 (6.8)
COVID-19 + Neurologic disease	6 (7.6)	2 (33.3)	4 (5.5)
Other (or Other +COVID-19)	6 (7.6)	0	6 (8.2)
**Disposition**			
Discharged	56 (70.8)	4 (66.6)	56 (76.7)
Deceased	21(26.5)	2 (33.3)	19 (26.0)
Still in hospital	2 (2.5)	0	2 (2.7)

*Acute intracranial hemorrhage includes subarachnoid, lobar, subdural, and subcortical/brainstem hemorrhages*.

*PRES, Posterior Reversible Encephalopathy Syndrome*.

*Worsening vasogenic edema occurred in the setting of multifocal metastatic disease*.

*“Other” reasons for admission: hip fracture (x2) +/– COVID-19; undifferentiated encephalopathy (COVID-19 negative on admission), falls in setting of decompensation, gastrointestinal cancer, and cardiac arrest*.

Seventy-six percent of the patients in our cohort were admitted primarily for SARS-CoV-2 PCR positivity and/or suspicion with associated symptoms, including acute respiratory distress syndrome, hypoxemia, cough, fever, and gastrointestinal illness. Two of these patients subsequently developed electrographic seizures. The other 4 patients with electrographic seizures were primarily admitted for acute neurologic illness (including refractory seizures and intracranial hemorrhage), three of whom had systemic COVID-19 symptoms or signs on admission (Table 1). Interestingly, one of these four patients was coincidentally found to have asymptomatic SARS-CoV-2 PCR positivity on admission with electrographic seizures observed within 24 h of admission.

Twenty-seven patients experienced acute neurological injury (ANI) either upon presentation or during hospitalization, with the most common causes being ischemic and hemorrhagic strokes (13.9 and 15.2% of study population, respectively; see Table 1). Only 7 patients in our study had a documented history of prior seizures, 3 of whom were subsequently found to have electrographic seizures. Four of the 6 patients with electrographic seizures in our cohort were discharged from the hospital while the other two suffered severe comorbid, fatal, neurologic disease (Table 1).

In patients who had seizures recorded on EEG, the interictal background was more likely to show focal slowing, sporadic interictal epileptiform discharges, and periodic epileptiform discharges. Specifically, four of the six patients with electrographic seizures had lateralized, generalized, or bilateral independent periodic epileptiform discharges. Each of these four patients had underlying brain pathology: multifocal brain metastases with worsening vasogenic edema, acute subarachnoid & intraparenchymal hemorrhage, prior subarachnoid hemorrhage complicated by epilepsy, and prior PRES with associated prior seizures. Of the other two patients with electrographic seizures, one had subdural and subarachnoid hemorrhage. The other had de Novo seizures without clear underlying brain pathology despite neurologic investigation (Tables 2, 3).

**Table 2:** EEG: Indication and findings.

**Indication for EEG**	**All patients (*n =* 79)**	**Seizures on EEG (*n =* 6)**	**No seizures on EEG (*n =* 73)**
Altered mental status	22 (27.8)	1 (16.7)	21 (28.8)
Acute hyperkinetic movements	30 (40.0)	5 (83.3)	25 (34.2)
Persistent coma	23 (29.1)	0	23 (31.5)
Other (including TTM)	4 (5.1)	0	4 (5.5)
**Findings on EEG**			
Diffuse slowing or attenuation	78 (98.7)	6 (100.0)	72 (98.6)
Focal slowing (not including bitemporal slowing)	11 (13.9)	4 (66.7)	7 (9.6)
Sporadic interictal ED	6 (7.6)	2 (33.3)	4 (5.5)
Periodic/Rhythmic ED	5 (6.3)	4 (66.7)	1 (1.4)
GPDs with triphasic morphology	11 (13.9)	0	11 (15.1)
NCSE	3 (3.8)	3 (50.0)	x

*ED, Epileptiform Discharges (not including GPDs with Triphasic Morphology); GPD, Generalized Periodic Discharges; NCSE, Non-Convulsive Status Epilepticus; TTM, Targeted Temperature Management*.

**Table 3:** Characteristics of patients with electrographic seizures.

**Patient #**	**Age**	**Gender**	**Primary indication for admission**	**Chronic neurological history**	**Acute neurological insult on admission or during hospital stay prior to seizures**	**Acute systemic COVID-19 symptoms on admission or during hospital stay prior to seizure**	**Reason for EEG connection**
A	51	M	Symptomatic COVID-19 infection (dyspnea and myocarditis) + witnessed new- onset seizures	N	N^*^	Y (dyspnea & myocarditis)	Hyperkinetic movements (left sided shaking)
B	57	F	Witnessed seizures + symptomatic COVID-19 & influenza infection	Y (multifocal metastatic lesions)	Y (worsening vasogenic edema on neuroimaging)	Y (febrile)	Hyperkinetic movements (clinical seizures)
C	61	F	Symptomatic COVID-19 infection	Y (PRES c/b focal epilepsy)	N	Y (severe hypoxia)	Hyperkinetic movements (myoclonic jerks)
D	68	F	Clinical status epilepticus	Y (ischemic stroke c/b focal epilepsy)	N	Y (febrile)	Hyperkinetic movements (clinical SE at OSH)
E	66	M	Symptomatic COVID-19 infection	N	Y (SDH & SAH)	Y (severe hypoxia)	Hyperkinetic movements (left arm/face shaking)
F	83	F	Acute intracranial hemorrhages with emergent craniotomy	N	Y (SAH, ICH, SDH)	N	Fluctuating mental status

* *Patient A was admitted for hyperkinetic movements in the setting of acute COVID-19 symptoms including dyspnea, myocarditis, and fever. Initial head CT scan did not show acute intracranial abnormality. However, subsequent imaging was not completed. This patient had widespread metastatic renal cell carcinoma, although there was no acute or prior evidence of intracranial metastases. Still, clinical suspicion was that electrographic seizures during this admission were triggered by unrecognized intracranial metastatic disease*.

Conversely, only one patient without electrographic seizures had periodic or rhythmic epileptiform activity; specifically, this patient developed lateralized periodic discharges over the left posterior head region in the setting of a poorly differentiated intraparenchymal process favored to be reversible posterior leukoencephalopathy over another inflammatory process.

Furthermore, the EEG was less likely to show generalized periodic discharges with triphasic morphology in patients who suffered electrographic seizures (Table 2).

## Discussion

We detected electrographic seizures in six of 79 patients with COVID-19 that underwent EEG monitoring, three of whom had new-onset seizures. This is a small percentage of patients suffering from electrographic seizures, and most were in the setting of hyperkinetic movements, acute neurologic disease on admission, and prior history of epilepsy or neurologic disease.

Specifically, three of the six patients with seizures on EEG had a prior history of seizures (see Table 3). Of these, one (patient B) had epilepsy secondary to brain metastases requiring dual anti-seizure therapy and had a breakthrough seizure 1 month prior to admission. A second patient (patient C) had epilepsy secondary to reversible posterior leukoencephalopathy related to liver transplantation and immunosuppression in 2017, which resulted in seizures and non-convulsive status epilepticus during the current admission. This patient continued to have breakthrough seizures every 3 months on triple therapy. A third patient (patient D) had epilepsy secondary to an ischemic infarct in 2012 and was seizure free on dual therapy prior to admission. All three patients initially had seizures similar to their known semiology, but all progressed to non-convulsive status epilepticus.

Of the remaining three patients (see Table 3), two (patients E & F) had concurrent intracranial hemorrhages—including subarachnoid hemorrhage; the latter is a known risk factor for seizures (11, 12). The final patient (patient A) had renal cell carcinoma which was widely metastatic, including metastases to the spine. A non-contrast head CT did not disclose any obvious intracranial abnormalities, but further workup for intracranial disease was not completed before discharge. He was transitioned to palliative care shortly after discharge due to systemic progression of malignancy. Although the patient was mildly hyponatremic on admission, the clinical suspicion upon discharge was that unrecognized intracranial metastatic disease was the most likely etiology for clinical and electrographic seizures (see Table 3).

In addition to history of seizures and brain injury/disease, we found that hyperkinetic movements and periodic or rhythmic epileptiform activity on EEG were more common in patients with electrographic seizures; no patients with persistent coma or GPDs with triphasic morphology suffered electrographic seizures. Of our patients connected to cvEEG for persistent coma in COVID-19, none experienced seizures. As such, the diagnostic yield of cvEEG in comatose COVID patients—without the mitigating risk factors discussed above—appears to be low.

There were several strengths to the study. First, cases were drawn from 4 hospitals within the same hospital network, which yielded a fairly large number of patients included in study. Moreover, all patients included in the study underwent EEG for a median of 2 days per patient. ACNS and Salzburg criteria were systematically applied to all EEGs reviewed, thus yielding standardized and relatively generalizable results across institutions.

The main limitation of this study is the lack of a SARS-CoV-2 negative comparator group to which these results could be evaluated. However, this case series was in part inspired by a similar study investigating the occurrence of electrographic seizures in critically ill patients at the same institution (8). Specifically, those investigators evaluated 98 patients admitted to Medical ICU for severe sepsis secondary to non-COVID-19 infections. They found 14 episodes of periodic discharges without non-convulsive seizures and 11 episodes of periodic discharges plus non-convulsive seizures. Direct comparison of these two studies is clearly limited, particularly given that 20% of the patients in this case series did not require intensive care. Still, in the cohort analysis by Gilmore and colleagues, they found prior history of neurologic disease to be a significant predictor of periodic discharges and non-convulsive seizures in their patients (8). Similarly, we found five of the 6 patients with electrographic seizures in this study to have chronic and/or new-onset neurologic disease. Another significant limitation of this study is the potential for under sampling given the need to reduce exposure to our EEG technicians.

Ultimately, we found a relatively low incidence of electrographic seizures (8%) in SARS-CoV-2 positive patients undergoing cvEEG monitoring. Five of the six patients with electrographic seizures had history of chronic and/or acute neurologic disease otherwise sufficient to cause or trigger seizures. As discussed above, one of the six patients with electrographic seizures had no clear history of chronic or acute neurological disease; however, the leading clinical suspicion was that unrecognized intracranial metastases were the most likely etiology for these seizures.

This adds to evolving literature indicating that seizures comprise a relatively small percentage of patients undergoing cvEEG monitoring in the setting of COVID-19 illness (13, 14). Moreover, findings on interictal EEG such as focal slowing and epileptiform discharges appear to increase the likelihood of also recording electrographic seizures in this population. If other studies support these findings, routine EEG may be sufficient to identify COVID-19 patients at risk of electrographic seizures without the mitigating factors discussed above. Given risk to staff and limited resources in COVID-19 pandemic, using the aforementioned risk factors—including hyperkinetic movements, chronic and/or acute intracranial disease, history of epilepsy, and epileptiform discharges or focal slowing on routine EEG—may help identify patients more appropriately of cvEEG monitoring.

## Data Availability Statement

The raw data supporting the conclusions of this article will be made available by the authors upon request, without undue reservation.

## Ethics Statement

The studies involving human participants were reviewed and approved by Columbia University Medical Center, Institutional Review Board. Written informed consent for participation was not required for this study in accordance with the national legislation and the institutional requirements.

## Author Contributions

BW, AM, and DB contributed to drafting the manuscript. BW, AM, DB, and AB contributed to data acquisition. BW, AM, and MB contributed to data analysis and preparing the table. All authors contributed to study design, critical review, and manuscript revision.

## Conflict of Interest

The authors declare that the research was conducted in the absence of any commercial or financial relationships that could be construed as a potential conflict of interest.
